# High Level Expression of a Novel Family 3 Neutral β-Xylosidase from *Humicola insolens* Y1 with High Tolerance to D-Xylose

**DOI:** 10.1371/journal.pone.0117578

**Published:** 2015-02-06

**Authors:** Wei Xia, Pengjun Shi, Xinxin Xu, Lichun Qian, Ying Cui, Mengjuan Xia, Bin Yao

**Affiliations:** 1 College of Animal Science, Zhejiang University, Hangzhou 310058, P. R. China; 2 Key Laboratory for Feed Biotechnology of the Ministry of Agriculture, Feed Research Institute, Chinese Academy of Agricultural Sciences, Beijing 100081, P. R. China; 3 Biotechnology Research Institute, Chinese Academy of Agricultural Sciences, Beijing 100081, P. R. China; Institute of Oceanology, Chinese Academy of Sciences, CHINA

## Abstract

A novel β-xylosidase gene of glycosyl hydrolase (GH) family 3, *xyl3A*, was identified from the thermophilic fungus *Humicola insolens* Y1, which is an innocuous and non-toxic fungus that produces a wide variety of GHs. The cDNA of *xyl3A*, 2334 bp in length, encodes a 777-residue polypeptide containing a putative signal peptide of 19 residues. The gene fragment without the signal peptide-coding sequence was cloned and overexpressed in *Pichia pastoris* GS115 at a high level of 100 mg/L in 1-L Erlenmeyer flasks without fermentation optimization. Recombinant Xyl3A showed both β-xylosidase and α-arabinfuranosidase activities, but had no hydrolysis capacity towards polysaccharides. It was optimally active at pH 6.0 and 60°C with a specific activity of 11.6 U/mg. It exhibited good stability over pH 4.0–9.0 (incubated at 37°C for 1 h) and at temperatures of 60°C and below, retaining over 80% maximum activity. The enzyme had stronger tolerance to xylose than most fungal GH3 β-xylosidases with a high *K_i_* value of 29 mM, which makes Xyl3A more efficient to produce xylose in fermentation process. Sequential combination of Xyl3A following endoxylanase Xyn11A of the same microbial source showed significant synergistic effects on the degradation of various xylans and deconstructed xylo-oligosaccharides to xylose with high efficiency. Moreover, using *p*NPX as both the donor and acceptor, Xyl3A exhibited a transxylosylation activity to synthesize *p*NPX_2_. All these favorable properties suggest that Xyl3A has good potential applications in the bioconversion of hemicelluloses to biofuels.

## Introduction

Hemicelluloses, mainly composed of hetero-1,4-β-d-xylans and hetero-1,4-β-d-mannans, are the second most abundant renewable polysaccharides in nature [1]. As the major component of hemicellulose, xylan is composed of a backbone of β-1,4-linked d-xylopyranosyl residues and side chains of different substituents. Thus, the degradation of xylan requires action of a battery of debranching and depolymerizing activities, which is achieved by the synergic hydrolysis of multiple enzymes. Specifically, xylanase attacks internal xylosidic linkages on the backbone, β-xylosidase releases xylosyl residues by endwise attack of xylooligosaccharides, and other debranching enzymes including α-l-arabinofuranosidase, α-glucuronidase and esterases remove branched chains [2]. Single xylosyl residues released from xylooligosaccharides and reducing end product inhibit xylanases, which makes β-xylosidase a key enzyme in the xylanolytic system with a great potential in many biotechnological applications.

β-Xylosidases (EC 3.2.1.37) have been classified into the glycoside hydrolase (GH) families 3, 39, 43, 52 and 54 based on the amino acid sequence similarities (http://www.cazy.org/Glycoside-Hydrolases.html) [3]. Their catalytic mechanisms are distinct; for instance, those of GH3 employ retaining catalysis and GH43 ones have inverting catalysis. Up to the present days, β-xylosidases have been described from a variety of microorganisms including bacteria and fungi, and fungal β-xylosidases are mostly grouped into GH3 [4–8]. Although β-xylosidase has been a research hotspot in the biofuel field in recent years due to its fermentation ability on pentose sugars [9], few fungal β-xylosidases of GH3 have yet been identified or heterologously expressed. Therefore, a better understanding of the deconstruction of xylan to constituent sugars, mainly xylose, for subsequent fermentation by ethanol producing microbes is critical to the efficient use of plant biomass for biofuel production [10].

Thermophilic and thermostable enzymes from thermophilic fungi are usually used in the conversion of hemicellulose to reduce the risk of contamination and increase the solubility of substrate [11]. Of the fungal sources, thermophilic *Humicola* is widely considered to be an innocuous and non-toxic fungus that produces a wide variety of GHs. Cellulases and hemicellulases from this genus have favorable characteristics and are utilized in various commercial applications, such as enzyme detergents (Novo Industri A/S., Enzymatic detergent additive, U.S. Patent 4810414) and enzyme preparations (Ultraflo L, Novozymes A/S, mainly composed of β-glucanase and xylanase). *Humicola insolens* Y1 has been reported to be an excellent producer of xylanolytic enzymes [12, 13], in which three thermophilic GH10 xylanases, one GH11 xylanase, and two bifunctional GH43 xylosidase/arabinosidases have been characterized. In this study, a novel β-xylosidase gene of GH3 was identified in *H*. *insolens* Y1 and overexpressed in *Pichia pastoris*. Biochemical characterization and synergy test with xylanase on xylan degradation were also carried out. This is a prior article for now through exhaustively describing the sequence identity, enzymatic properties, xylose tolerance, transxylosylation activity and synergistic action with xylanase of a fungal GH3 β-xylosidase.

## Materials and Methods

### Strains, media, vectors and chemicals

*H*. *insolens* Y1 GCMCC 4573 was routinely cultured in wheat bran medium as described previously [12]. *Escherichia coli* Trans1-T1 and vector pEASY-T3 (TransGen, Beijing, China) were used for gene cloning. The heterologous protein expression system of pPIC9 and *P*. *pastoris* GS115 (Invitrogen, Carlsbad, CA) was employed as the gene expression vector and expression host, respectively. The DNA purification kit, restriction endonucleases and *LA Taq* DNA polymerase were purchased from TaKaRa (Otsu, Japan). T4 DNA ligase and the total RNA isolation system kit were purchased from Promega (Madison, WI). The cDNA synthesis kit was purchased from TransGen.

Beechwood xylan, 4-nitrophenyl β-d-xylopyranoside (*p*NPX), 4-nitrophenyl α-l-arabinofuranoside (*p*NPAf), 4-nitrophenyl α-d-galactopyranoside (*p*NPGal), 4-nitrophenyl β-d-glucopyranoside (*p*NPG), 4-nitrophenyl α-l-arabinopyranoside (*p*NPAb) and 4-nitrophenyl β-d-cellobioside (*p*NPC) were purchased from Sigma-Aldrich (St. Louis, MO). Soluble wheat arabinoxylan was obtained from Megazyme (Wicklow, Ireland). All other chemicals used were of analytical grade and commercially available.

### Cloning of the cDNA of *xyl3A*

Total RNA of *H*. *insolens* Y1 was extracted from the mycelia after 3 days’ growth in the inducing medium [12], and was reverse transcribed into cDNA by RT-PCR following the protocol of One-Step gDNA Removal and cDNA Synthesis SuperMix (TransGen). Based on the partial genome sequence of strain Y1 (whole genome sequencing in progress), a β-xylosidase encoding gene of GH3, *xyl3A*, was identified, and the gene fragment without the signal peptide-coding sequence was amplified with the specific primer set (Xyl3A-F: 5′-GGGGAATTCGGGGTCCTCCCCGACTGCAGCAAGCCG-3′, and Xyl3A-R: 5′-GGGGCGGCCGCTCAATTCTTGAGGTTGGGGTCAACAG-3′, restriction sites underlined). *H*. *insolens* Y1 cDNA was used as the template, and the annealing temperature was 60°C. The PCR products were purified and ligated into the pEASY-T3 vector for sequencing.

### Sequence analysis

DNA and protein sequence were aligned using the BLASTn and BLASTp programs (http://www.ncbi.nlm.nih.gov/BLAST/), respectively. Vector NTI Advance 10.0 software (Invitrogen) was used to analyze DNA sequence and to predict the molecular weight of protein. Genes, introns, exons and transcription initiation sites were predicted using the online software FGENESH (http://linux1.softberry.com/berry.phtml). Multiple sequence alignments were performed with the ClustalW software. The signal peptide and the potential *N*-glycosylation sites were predicted by the SignalP 4.1 server (http://www.cbs.dtu.dk/services/SignalP/) and the NetNGlyc 1.0 Server (http://www.cbs.dtu.dk/services/NetNGlyc/), respectively.

### High level expression and purification of recombinant Xyl3A

The cDNA of *xyl3A* without the signal peptide-coding sequence and the pPIC9 vector were both digested by *EcoR*I and *Not*I and ligated into in-frame fusion of the α-factor signal peptide to construct the recombinant plasmid pPIC9-*xyl3A*. The recombinant plasmid was linearized using *Bgl*II and transformed into *P*. *pastoris* GS115 competent cells by electroporation using Gene Pulser X cell Electroporation System (Bio-Rad, Hercules, CA). Minimal dextrose medium (MD) plates were prepared for the screening of positive transformants. The positive transformants were transferred to buffered glycerol complex medium (BMGY) and grown at 30°C for 2 days. The cells were collected by centrifugation and resuspended in buffered methanol complex medium (BMMY) containing 0.5% methanol for induction. The β-xylosidase activities of the culture supernatants were assayed as described below, and the transformant exhibiting the highest β-xylosidase activity was subjected to high level expression in 1-L Erlenmeyer flasks according to the *Pichia* expression manual (Invitrogen).

The culture supernatants of aforementioned recombinant strain were collected by centrifugation (12,000 × *g*, 4°C, 10 min) to remove cell debris and undissolved materials, followed by concentration through a Vivaflow ultrafiltration membrane (Vivascience, Hannover, Germany) with a molecular weight cut-off of 5 kDa. The crude enzyme was loaded onto a FPLC HiTrap Q Sepharose XL 5 mL column (GE Healthcare, Uppsala, Sweden) that was equilibrated with 20 mM Tris-HCl (pH 8.0). Proteins were eluted using a linear gradient of NaCl (0–1.0 M) in the buffer mentioned above at a flow rate of 3.0 mL/min. Fractions exhibiting β-xylosidase activities were pooled and subjected to sodium dodecyl sulfate-polyacrylamide gel electrophoresis (SDS-PAGE). The protein concentration was determined by a Bradford assay with bovine serine albumin as a standard. To remove *N*-glycosylation during heterologous expression in *P*. *pastoris*, purified recombinant Xyl3A was incubated with 500 U of endo-β-*N*- acetylglucosaminidase H (Endo H) at 37°C for 2 h according to the manufacturer’s instructions (New England Biolabs, Ipswich, MA). The deglycosylated enzyme was also analyzed by SDS-PAGE.

### Enzyme activity assay

The β-xylosidase activity was assayed using *p*NPX as the substrate. The standard reaction system consisted of 250 μL of appropriately diluted enzyme and 250 μL of McIlvaine buffer (pH 6.0) containing 2 mM *p*NPX. After incubation at 60°C for 10 min, 1.5 mL of 1.0 M Na_2_CO_3_ was added into the system to terminate the reaction. The amount of *p*-nitrophenol released was determined spectrophotometrically by reading the absorbance at 405 nm. One unit of β-xylosidase activity was defined as the amount of enzyme that released 1 μmol of *p*-nitrophenol per minute under the assay conditions. Each experiment was performed in triplicate.

### Biochemical characterization

The optimal pH for Xyl3A activity was determined at 60°C for 10 min over a pH range of 2.0–11.0 using the following buffers: 100 mM Na_2_HPO_4_-citric acid (pH 2.0–8.0), 100 mM Tris-HCl (pH 8.0–9.0), and 100 mM glycine-NaOH (pH 9.0–11.0). To estimate pH stability, the enzyme was pre-incubated in the buffers mentioned above without substrate at 37°C (physiological temperature) or 60°C (optimal temperature) for 1 h, and the residual activities were measured under the standard conditions (pH 6.0, 60°C, 10 min).

The optimal temperature was examined at the optimal pH by measuring the enzyme activity over the temperature range of 30 and 90°C. The thermostability was investigated by determining residual enzyme activities after preincubation at 60°C or 70°C and optimal pH without substrate for various periods. The samples were collected at 0, 2, 5, 10, 20, 30 and 60 min, respectively. Enzyme activity assays were performed under the standard conditions.

The enzyme activity of Xyl3A was measured in the presence of 5 mM of various metal ions and chemical reagents (Ag^+^, Ca^2+^, Li^+^, Co^2+^, Cr^3+^, Ni^2+^, Cu^2+^, Mg^2+^, Fe^3+^, Mn^2+^, Hg^2+^, Pb^2+^, EDTA, SDS or β-mercaptoethanol) to estimate their effect on Xyl3A activity. The reaction without any additive was used as a blank control.

### Substrate specificity and kinetic parameters

To investigate the substrate specificity of Xyl3A, activity assays were performed under optimum conditions with 2 mM of *p*-nitrophenyl derivatives (*p*NPX, *p*NPAf, *p*NPG, *p*NPGal, *p*NPAb, *p*NPC) or 1% (w/v) polysaccharides (beechwood xylan, water-soluble wheat arabinoxylan, sugar beet arabinan and debranched AZCL-arabinan) as the substrate.

The *K*_*m*_, *V*_*max*_ and *K*_*cat*_ values of Xyl3A were determined at pH 6.0 and 60°C for 5 min in 100 mM Na_2_HPO_4_-citric acid containing 1–10 mM *p*NPX as the substrate. The data were plotted according to the Lineweaver-Burk method.

### Xyl3A tolerance to xylose

The tolerance of Xyl3A to xylose was investigated as described by Yan et al. [14]. The β-xylosidase activity of Xyl3A in each reaction system containing xylose of different concentrations (5–50 mM) was determined in the presence of two final concentrations of *p*NPX (0.75 mM and 1 mM). The residual β-xylosidase activities were measured according to the standard assay method. The data were analyzed by a Dixon plot. The *K*_*i*_ value of xylose was defined as the amount of xylose required to inhibit 50% of the Xyl3A activity.

### Transxylosylation of Xyl3A

For transxylosylation assay, *p*NPX was added to serve as both the donor and acceptor. The reaction system containing 75 mM of *p*NPX and 3 U/mL of Xyl3A was incubated at pH 6.0 and 37°C for 0–24 h. After the removal of Xyl3A by ultrafiltration, the transxylosylation products were analyzed by thin layer chromatography (TLC) using the mixture of xylose, xylobiose and xylotriose as standards. TLC was performed on aluminum-coated silica gel 60 sheets (Merck, Darmstadt, Germany), developed in a system of *n*-butyl alcohol/acetic acid/water 2:1:1 (v/v/v), and colored by saturating the plate in 5% sulphuric acid (v/v) and then heating at 110°C for 5 min.

### Enzyme synergy

Xyl3A and Xyn11A of the same microbial source were selected to study their synergic actions on the hydrolysis of beechwood xylan and water soluble wheat arabinoxylan [13]. Basic manipulation consulted to the method reported by Raweesri et al. [15]. Each reaction containing 900 μL of 0.5% (w/v) substrate and 100 μL of enzyme(s) in 100 mM Na_2_HPO_4_-citric acid (pH 6.0) was incubated at 37°C for 12 h. Reactions containing substrate only were treated as blank controls. Enzymes, 0.25 U of Xyl3A and/or 0.25 U of Xyn11A, were added alone, simultaneously or sequentially. For sequential reactions, the first reactions were terminated by 10-min boiling water bath, and the second reactions were performed as described above. The xylose equivalents (mM) released were determined using the DNS method at room temperature, with xylose as the standard.

The synergy degree was defined as the ratio of xylose equivalents released by enzyme combinations to the sum of the xylose equivalents released by each enzyme alone. One-way ANOVA with a Tukey’s test was used for statistical analysis in OriginPro 8.

## Results and Discussion

### Gene cloning and sequence analysis

The full-length cDNA of *xyl3A* from *H*. *insolens* Y1 contains 2334 bp and encodes a polypeptide of 777 amino acid residues and a termination codon. Deduced Xyl3A consists of a putative signal peptide at the N-terminus (residues 1–19) and a catalytic domain of GH3 (residues 20–777). The calculated molecular mass and *p*I value were estimated to be 83.2 kDa and 6.21, respectively. The *p*I value of Xyl3A is higher than that of most fungal β-xylosidases (4.0–5.0) [16]. Five potential *N*-glycosylation sites (Asn110, Asn252, Asn314, Asn555, and Asn570) were identified in deduced Xyl3A.

**Fig 1:**
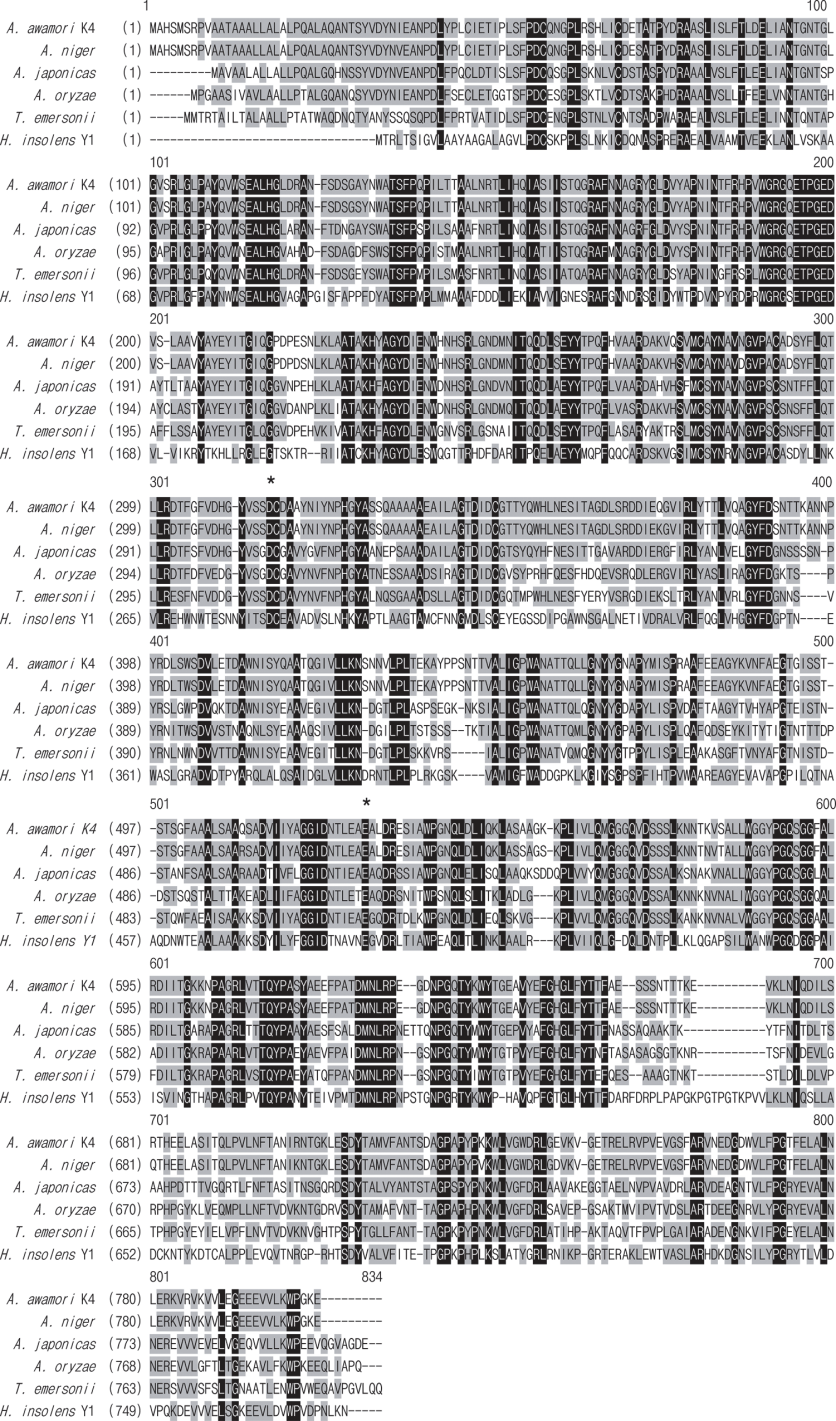
Sequence alignment of deduced Xyl3A from *H*. *insolens* Y1 with other GH3 β-xylosidases from *A*. *awamori* K4 (Q4AEG8), *A*. *nidulans* (CAA73902), A. *oryzae* (T00392), *A*. *niger* (CAB06417) and *T*. *emersonii* (JC7966). Identical and similar residues are shaded in black and grey, respectively. The putative catalytic residues are marked with asterisks.

Deduced Xyl3A exhibits highest sequence identities of 64% with a putative (trans)glycosidase from *Glarea lozoyensis* ATCC 20868 and relative low sequence identities with functionally characterized GH3 β-xylosidases (Fig. 1), i.e. 41% with Xyl from *Aspergillus awamori* K4 [4] and XylA from *Aspergillus japonicas* [5], 40% with XlnD from *Aspergillus nidulans* [6], Bxl1 from *Talaromyces emersonii* [7] and XylA from *Aspergillus oryzae* [8]. Blast analysis against the PDB database indicates that Xyl3A shares a fairly low sequence similarity to crystal-resolved counterparts (29% identity the highest), thus no available template was found to predict the tertiary structure of Xyl3A.

The catalysis mechanism of GH3 β-xylosidases has been proved to proceed via a retaining reaction mechanism under the operation of a nucleophile Glu and an acid/base residue Asp. Based on the amino acid sequence alignments, the two putative catalytic residues of Xyl3A were predicted to be Asp282 and Glu488, corresponding to Asp307 and Glu516 of XylA from *A*. *japonicas* [5] and Asp242 and Glu441 of Bxl1 from *T*. *emersonii* [7].

### Overexpression and purification of recombinant Xyl3A

Bacterial enzymes are generally heterologously expressed in *E*. *coli* [17], while that of fungi are usually purified from original cultures [18] or produced in high-level expression systems like *P*. *pastoris* [19] or *Aspergillus* [8]. In this study, we successfully overexpressed mature Xyl3A without the signal peptide in *P*. *pastoris* GS115 with methanol induction, which was secreted into culture supernatant by the α-factor signal peptide. Without fermentation optimization, the yield of Xyl3A reached approximately 100 mg/L. The result suggests its great potential for cost-saving mass production.

**Fig 2:**
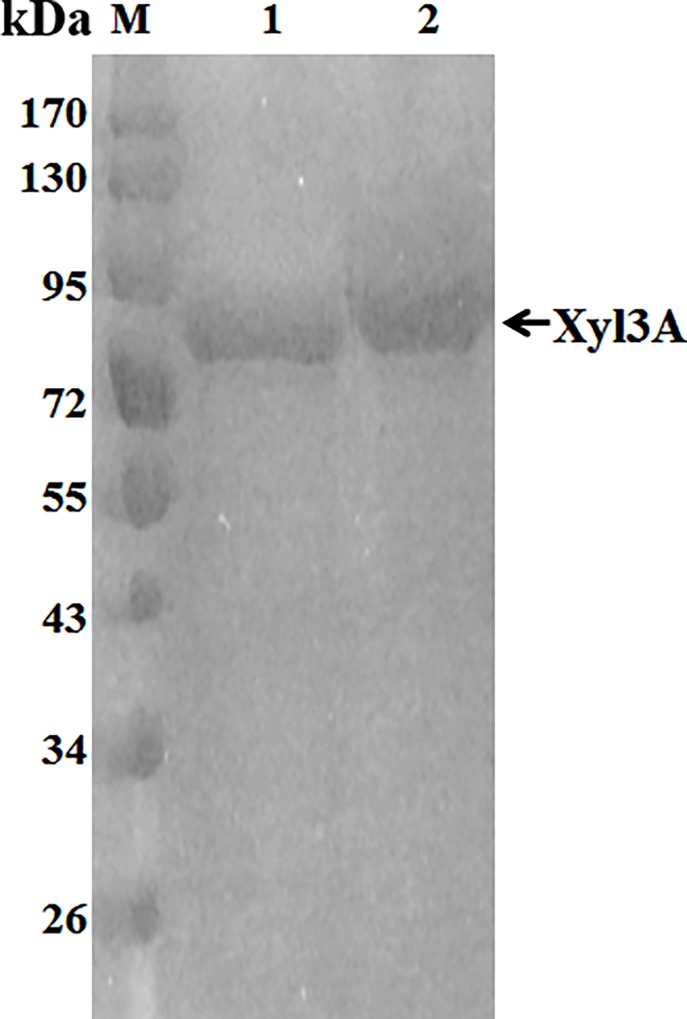
SDS-PAGE analysis of the purified recombinant Xyl3A. Lanes: 1, the purified recombinant Xyl3A after deglycosylation with Endo H; 2, the purified recombinant Xyl3A; M, the standard protein molecular weight markers.

Crude Xyl3A was concentrated by nearly ten fold in volume and purified by a one-step anion exchange chromatography. The purified enzyme solution exhibited 8.1 U/mL of β-xylosidase activity under its optimum condition with a concentration of 0.698 mg/mL. Purified Xyl3A migrated one single band of about 85.0 kDa on SDS-PAGE (Fig. 2). After deglycosylation with Endo H, Xyl3A showed a slight decrease in molecular mass, which was in agreement with the calculated molecular weight. Similar molecular masses have been reported for some GH3 β-xylosidases [4, 18]. Mozolowski and Connerton [20] purified a GH3 β-xylosidase from *H*. *insolens* culture, which was composed of two protein subunits of 64 kDa and 17 kDa with a total molecular mass of approximately 81 kDa. Deduced Xyl3A showed high sequence identities (91–100%) to the digested peptide fragments of both subunits, but no supposed fracture occurred at the C-terminus of recombinant Xyl3A when heterologously expressed in *P*. *pastoris* GS115.

### Enzymatic properties of purified recombinant Xyl3A

**Fig 3:**
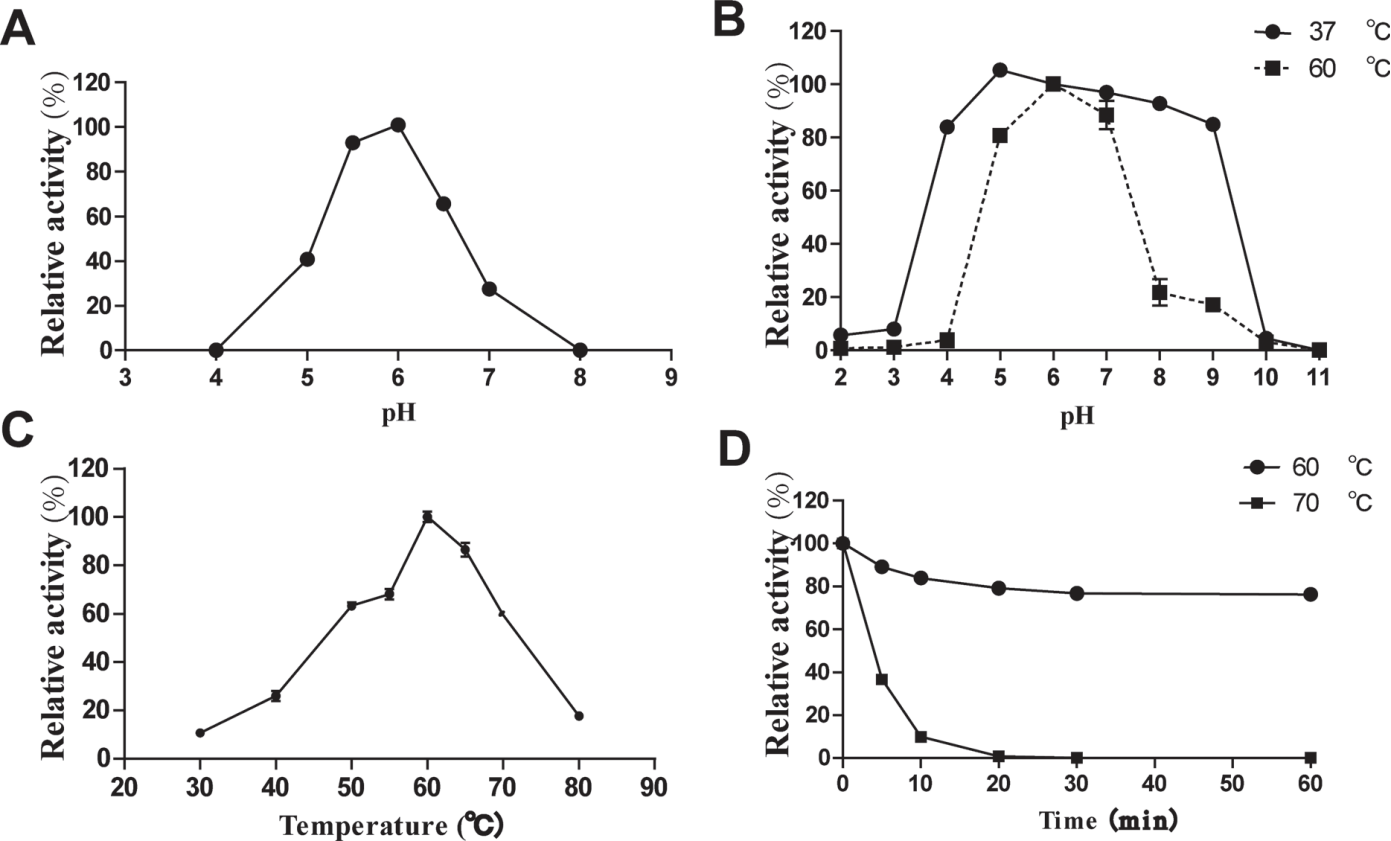
Properties of purified recombinant Xyl3A. (A) Effect of pH on Xyl3A activities. (B) pH stability of Xyl3A. (C) Effect of temperature on Xyl3A activities. (D) Thermostability of Xyl3A. Each value in the panel represents the means ± SD (n = 3).

Similar to the two GH43 β-xylosidases of *H*. *insolens* Y1 [13] but different from other fungal β-xylosidases that function at pH 3.5–5.0 (Table 1), purified recombinant Xyl3A was active in weak acid pH, showing optimal activity at pH 6.0, 65.5% activity at pH 6.5 and 27.4% at pH 7.0 (Fig. 3A). The pH stability of Xyl3A was assayed at both 37°C and 60°C (Fig. 3B). The enzyme had good pH stability at 37°C, retaining more than 80% activity after incubation at pH 4.0–9.0 for 1 h. After pre-incubation at 60°C for 1 h, the enzyme showed a narrower pH stability range (pH 5.07.0). When assayed Xyl3A activity at pH 6.0 (the optimal pH), it acted the best at a relatively high temperature of 60°C and remained 60% activity even at 70°C (Fig. 3C). This temperature optimum falls within the range of other fungal GH3 β-xylosidases (50–70°C; Table 1), but higher than that of β-xylosidase from *Neocallimastix frontalis* [21]. Xyl3A was thermostable at 60°C, retaining about 80% activity after 1 h incubation at 60°C without substrate, and lost activity rapidly when treated at 70°C (Fig. 3D). The enzymatic properties of *Humicola* GHs are much similar [12, 14, 20, 22], which might be due to their grown surroundings of neutral pH and high temperature.

**Table 1:** Property comparison of Xyl3A from *H*. *insolens* Y1 with other fungal β-xylosidases.

Species	MW (kDa)	Optimum activity		Specific activity (U/mg)	*K*_*m*_ (mM)	*V*_*max*_ (μmol/min/mg)	*K*_*i*_ (mM)	References
		pH	Temperature (°C)					
*Humicola insolens* Y1^a^	83.2	6.0	60	11.6	2.51	37.33	29	This work
*H*. *insolens* Y1^b^	37.0	6.5	50	20.5	12.2	203.8	79	[14]
*H*. *insolens* Y1^b^	62.0	7.0	50	1.7	1.29	2.18	292	[14]
*H*. *insolens* ^c^	68	5.5	70	193	1.74	22.17	-	[20]
*Aspergillus nidulans* ^c^	85	5.0	50	107.1	1.1	25.6	-	[6]
*Aureobasidium pullulans* ATCC 20524^c^	88.5	3.5	70	288	3.5	263	-	[18]
*Aspergillus awamori* K4^c^	84.6	4.0	70	19.58	-	-	-	[4]
*Aspergillus oryzae* ^d^	110	4.0	60	76	-	-	-	[8]
*Aspergillus phoenicis* ^c^	132	4.0–4.5	75	820.6	2.36	920.75	-	[24]
*Fusarium proliferatum* ^c^	91.2	4.5	60	53	0.77	75	5	[29]
*Neocallimastix frontalis* ^c^	90.0	6.5	35	4.3	122	-	-	[21]
*Penicillium herquei* IFO 4674^c^	103.7	4	50	353.6	0.12	-	-	[25]
*Talaromyces thermophilus* ^c^	97	7	50	147.5	2.37	0.049	-	[26]
*Trichoderma koningii* G-39^c^	104	3.5–4.0	55–60	3.02	0.04	-	5	[23]

^a^ Expressed in *P*. *pastoris* GS115.

^b^ Belonging to GH43 and expressed in *E*.*coli*.

^c^ Purified from the original fungi.

^d^ Expressed in *A*. *oryzae*.

The effects of metal ions and chemical reagents on the activity of Xyl3A were determined at the concentrations of 5 mM (Table 2). Xyl3A was highly resistant to EDTA, β-mercaptoethanol and almost all of tested cations except for Ag^+^ and SDS. Mn^2+^ enhanced the activity slightly.

**Table 2:** Effect of metal ions and chemical reagents (5 mM) on the activity of purified Xyl3A.

Chemicals	Relative activity (%)^a^	Chemicals	Relative activity (%)^a^
Control	100.0 ± 0.4	Mn^2+^	114.9 ± 0.2
Ag^+^	5.3 ± 0.2	Fe^3+^	83.9 ± 1.9
Li^+^	103.5 ± 0.4	Ni^2+^	99.5 ± 0.2
Pb^2+^	95.3 ± 0.3	Mg^2+^	94.7 ± 0.1
Ca^2+^	101.8 ± 0.3	Zn^2+^	93.4 ± 0.4
Cu^2+^	93.4 ± 0.3	EDTA	87.5 ± 0.2
Cr^3+^	94.4 ± 1.1	SDS	46.4 ± 0.2
Co^3+^	94.9 ± 0.4	β-Mercaptoethanol	92.0 ± 1.1
Co^3+^	94.9 ± 0.4		

^a^ Values represent the means of triplicates relative to the untreated control samples.

### Substrate specificity and kinetic parameters

Xyl3A exhibited the highest activity towards *p*NPX (100%), moderate on *p*NPAf (26.9%) and weak on *p*NPGal (2.57%). It has been reported that GH3 β-xylosidases have broad substrate specificity. For example, when the activity against *p*NPX was defined as 100%, β-xylosidase from *Asperigus pullulans* ATCC20524 showed 35.8% relative activity towards *p*NPAf [5], and β-xylosidase from *A*. *japonicus* strain MU-2 showed 31.6% β-glucosidase activity and 17.7% α-arabinfuranosidase activity [18]. However, the 64 kDa subunit of *H*. *insolens* β-xylosidase had no activity towards *p*NPAf [20], which was probably due to the deletion of the C-terminus. No activity was detected when using other polysaccharides as the substrate. In contrast, several bifunctional GH43 β-xylosidase-arabinosidases have higher α-arabinfuranosidase and xylanase activities [13]. The underlying mechanism might be ascribed to the spatial similarity of d-xylopyranose and l-arabinofuranose [23].

Using *p*PNX as the substrate, the specific activity of purified recombinant Xyl3A was determined to be 11.6 U/mg, which was higher than that of GH3 β-xylosidases from *Neocallimastix frontalis* (4.3 U/mg) [21] and *Trichoderma koningii* G-39 (3.02 U/mg) [23], but lower than that from *Aspergillus phoenicis* (820.6 U/mg) [24] and *Penicillium herquei* IFO 4674 (353.6 U/mg) [25]. The same conclusion applied to the comparison among four β-xylosidases produced by *H*. *insolens* [13, 20].

The *K*_m_ and *V*_max_ values of purified recombinant Xyl3A were 2.51 mM and 37.33 μmol/min/mg, respectively, when *p*NPX was used as the substrate. The *K*_m_ value of Xyl3A is similar to that of β-xylosidases from *Talaromyces thermophiles* (2.37 mM) [26] and *A*. *phoenicis* (2.36 mM) [24], suggesting its moderate affinity to substrate. In comparison with the kinetic values of other *H*. *insolens* isoenzymes (Xyl43A, 12.2 mM and 203.8 μmol/min/mg; Xyl43B, 1.29 mM and 2.18 μmol/min/mg; a 64 kDa β-xylosidase subunit, 1.74 mM and 22.17 μmol/min/mg), Xyl3A had a middle affinity and *V*_max_ towards *p*NPX.

### Xyl3A tolerance to xylose

d-Xylose is the principal end-product of xylooligosaccharide hydrolysis. It performs competitive inhibition on the activity of β-xylosidase. Thus a xylose-tolerant β-xylosidase is essential to maintain the efficiency of hemicellulose conversion by removing xylobioses, the natural substrate of β-xylosidase [27]. Based on the Dixon plot, Xyl3A had a *K*_*i*_ value of 29 mM, which is significantly higher than almost all reported fungal GH3 β-xylosidases (2 to 10 mM) [23, 28, 29]. Higher tolerance to xylose has been reported in fungal GH43 xylosidases, such as 79 mM and 292 mM for Xyl43A and Xyl43B of *H*. *insolens* Y1, respectively [13] and 139 mM for PtXyl43 [17]. However, the GH3 TthXynB3 from archaebacterium *Thermotoga thermarum* has an extremely high *K*_*i*_ value of 1 M [30]. Moreover, the enzymatic activity was even activated by xylose at concentrations less than 500 mM.

### Transxylosylation of Xyl3A

**Fig 4:**
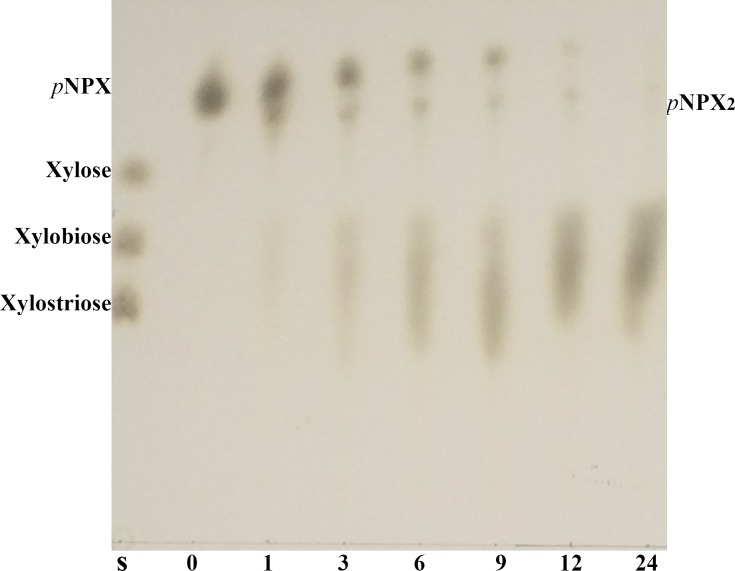
TLC analysis of the transxylosylation products of Xyl3A with *p*PNX as both the donor and acceptor. S, the xylooligosaccharide standards; 0–24, the time course of transxylosylation (h).

Preparation of useful oligosaccharides and glycosyl compounds has attracted broad attention in recent years. Compared with chemical approach, transglycosylation activities of glycosidases have advantages on the high specificity for substrate, the selectivity of the bonding, and the mildness of reaction conditions. Therefore transglycosylation ability has been regarded as an important characteristic of a glycosidase for potential applications [31]. Several studies have addressed the transxylosylation capacities of several GH3 β-xylosidases with oligosaccharides, alcohols and even *p*NPX as acceptors [32–34]. In the present study, the transxylosylation ability of Xyl3A was determined by TLC (Fig. 4). After co-incubation of Xyl3A and *p*NPX for 1 h, two products appeared on the TLC plate, which are conjectured to be *p*NPX_2_ and xylobiose. Along with time, the amount of xylobiose increased while that of *p*NPX_2_ decreased. The possible reason might be that *p*NPX acts as the primary xylosyl acceptor when the concentration of xylose is relatively low in the early stage of the reaction, but is slowly hydrolyzed when *p*NPX runs out. As the time went on, *p*NPX_2_ was hydrolyzed, and the released xylosyl residues formed xylobiose in turn. No larger oligosaccharide or *p*NP-oligosaccharide was observed, indicating that transxylosylation by Xyl3A tended to produce short chain oligosaccharides with low polymerization degree. It was quite different from *Aspergillus* β-xylosidase that was able to catalyze the synthesis of *p*-nitrophenyl (*p*NP)-(1,4)-d-xylooligosaccharides with a degree of polymerization of 2–7 [33].

### Xylan degradation by enzyme synergy

β-Xylosidase is usually used in combination with xylanase to fully deconstruct hemicelluloses and β-1,4-xylooligosaccharides, which products can be fermented to biofuels like ethanol and butanol [35]. In the current study, a synergy value was used to quantify the cooperation between Xyl3A and Xyn11A (Table 3). Significant differences (*p* < 0.05) were observed among different enzyme combinations on xylan degradation. Birchwood xylan with less side chains acted as a better substrate for synergism. No matter which substrate was used, the sequential enzyme combination of Xyn11A followed by Xyl3A exhibited the highest synergy degrees (1.25 and 1.08, respectively), and Xyl3A alone released the least xylose equivalents. This result suggests that Xyl3A works better on xylooligosacchardies rather than on xylan polysaccharides.

**Table 3:** Synergetic reactions by Xyn11A and Xyl3A against two xylan substrates.^a^

Enzyme added		Beechwood xylan^b^		Wheat arabinoxylan^c^	
First reaction	Second reaction	Xylose equivalents (mM)	Synergy ^d^	Xylose equivalents (mM)	Synergy
Xyn11A	None	6.63 ± 0.13	-	6.36 ± 0.06	-
Xyl3A	None	0.12 ± 0.06	-	0.06 ± 0.04	-
Xyn11A+Xyl3A	None	7.63 ± 0.09	1.13^*^	6.08 ± 0.02	0.95^*^
Xyn11A	Xyl3A	8.42 ± 0.03	1.25^*^	6.94 ± 0.23	1.08^*^
Xyl3A	Xyn11A	7.95 ± 0.13	1.18^*^	6.80 ± 0.14	1.06^*^

^a^ Simultaneous reactions refer to the reactions with two enzymes added simultaneously; sequential reactions refer to the reactions with enzymes added sequentially.

^b^ Beechwood xylan:the arabinose:xylose ratio of ∼1:90.

^c^ Water-soluble wheat arabinoxylan: the arabinose:xylose:other sugars ratio of ∼37:61:2.

^d^ Synergy degree is defined as the ratio of xylose equivalents from enzyme combinations to the sum of that released by the individual enzymes;

the data marked with^*^ means significant difference at *p* < 0.05 (One-way ANOVA with a Tukey’s test by OriginPro 8).

In comparison with Xyl43A and Xyl43B of *H*. *insolens* Y1 [13], Xyl3A displayed almost equal synergistic effects with xylanase at a quarter of enzyme dosage. Considering the absence of standard signal peptides in Xyl43A and Xyl43B, Xyl3A may act as the primary extracellular β-xylosidase for xylan degradation in *H*. *insolens* Y1 under natural conditions.

## Conclusions

A novel GH3 β-xylosidase gene was identified in *H*. *insolens* Y1, and its gene fragment without the signal peptide-coding sequence was overexpressed in *P*. *pastoris* GS115 with a high level of 100 mg/L. Recombinant Xyl3A exhibited β-xylosidase, α-arabinosidase and transxylosylation activities. Using *p*PNX as the substrate, the enzyme had maximum activity at pH 6.0 and 60°C and retained stable over pH 4.0−9.0 (after incubation at 37°C for 1 h) and at temperatures of 60°C and below. Moreover, it was highly tolerant to xylose (up to 29 mM) and had significant synergistic action with xylanase on the degradation of various xylans. These properties make Xyl3A prospective for application in the bioconversion of hemicellulose and biofuel industry.
